# Virological surveillance of influenza viruses in the WHO European Region in 2019/20 – impact of the COVID-19 pandemic

**DOI:** 10.2807/1560-7917.ES.2020.25.46.2001822

**Published:** 2020-11-19

**Authors:** Angeliki Melidou, Dmitriy Pereyaslov, Olav Hungnes, Katarina Prosenc, Erik Alm, Cornelia Adlhoch, James Fielding, Miriam Sneiderman, Oksana Martinuka, Lucia Pastore Celentano, Richard Pebody, WHO European Region influenza surveillance network, WHO European Region Influenza Surveillance Network author list, WHO European Region Influenza Surveillance Network author list, Monika Redlberger-Fritz, Judith Aberle, Ramona Trebbien, Jakob Nybo Nissen, Niina Ikonen, Anu Haveri, Ralf Dürrwald, Georgia Gioula, Maria Exindari, Athanasios Kossyvakis, Andreas Mentis, Linda Dunford, Lisa Domegan, Maria Rita Castrucci, Simona Puzelli, Natalija Zamjatina, Gatis Pakarna, Algirdas Griskevičius, Asta Skrickiene, Guillaume Fournier, Joel Mossong, Adam Meijer, Ron Fouchier, Karoline Bragstad, Olav Hungnes, Raquel Guiomar, Pedro Pechirra, Mihaela Lazar, Cherciu Carmen Maria, Andrey Komissarov, Daria Danilenko, Elena Burtseva, Elena Tichá, Edita Staronova, Katarina Prosenc, Nataša Berginc, Francisco Pozo, Majadahonda Inmaculada Casas, Mia Brytting, Ana Rita Gonçalves, Iryna Demchyshyna, Angie Lackenby, Catherine Thompson, Rory N Gunson, Samantha J Shepherd, Catherine Moore, Simon Cottrell, Angeliki Melidou, Cornelia Adlhoch, Oksana Martinuka, Pasi Penttinen, Lucia Pastore Celentano, Dmitriy Pereyaslov, Miriam Sneiderman, James Fielding, Richard Pebody, John McCauley, Rodney Daniels

**Affiliations:** 1 European Centre for Disease Prevention and Control (ECDC), Stockholm, Sweden; 2 World Health Organization (WHO) Regional Office for Europe, Copenhagen, Denmark; 3 Norwegian Institute of Public Health, Oslo, Norway; 4 Laboratory for Public Health Virology, National Influenza Centre Slovenia, National Laboratory for Health, Environment and Food, Ljubljana, Slovenia; 5 The members of the network are listed below

**Keywords:** surveillance, influenza virus, SARS-CoV-2, virus characterization, COVID-19, pandemic, Europe

## Abstract

The COVID-19 pandemic negatively impacted the 2019/20 WHO European Region influenza surveillance. Compared with previous 4-year averages, antigenic and genetic characterisations decreased by 17% (3,140 vs 2,601) and 24% (4,474 vs 3,403). Of subtyped influenza A viruses, 56% (26,477/47,357) were A(H1)pdm09, 44% (20,880/47,357) A(H3). Of characterised B viruses, 98% (4,585/4,679) were B/Victoria. Considerable numbers of viruses antigenically differed from northern hemisphere vaccine components. In 2020/21, maintaining influenza virological surveillance, while supporting SARS-CoV-2 surveillance is crucial.

The ending of the 2019/20 influenza season in the World Health Organization (WHO) European Region coincided with the start of the first wave of the coronavirus disease (COVID-19) pandemic. This study assesses potential impacts of the pandemic on influenza surveillance and presents characteristics of influenza viruses detected in the Region in 2019/20, relative to contemporary components of influenza vaccines for the northern hemisphere (NH).

## Influenza virological surveillance in Europe, influenza season 2019/20

**Figure 1:**
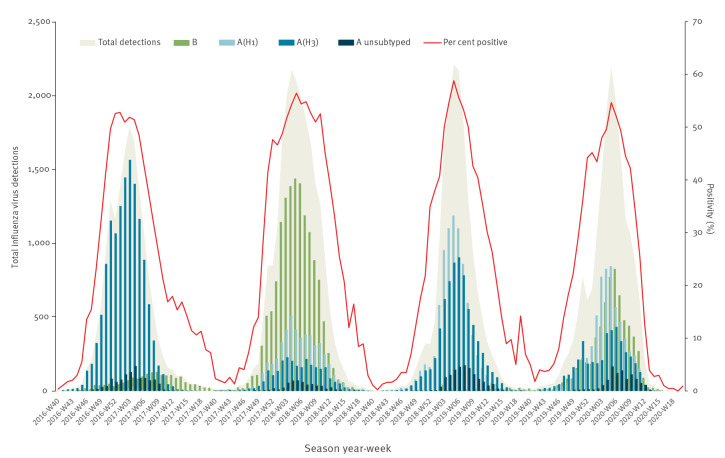
Number of positive sentinel specimens and positivity by week of reporting, week 40 2016 to week 20 2020, over four consecutive seasons, WHO European Region, 2016/17–2019/20

In the WHO European Region, the 2019/20 influenza season started in week 47 2019, peaked for 2 weeks, weeks 05 and 06 2020, and returned to baseline levels (< 10% positivity in sentinel samples) very rapidly in week 13 2020, following widespread public health and social measures implemented to control COVID-19 (Figure 1). Influenza type A viruses (120,493; 72.9%) dominated over type B (44,774; 27.1%). Of 47,357 subtyped influenza A viruses, 26,477 (56%) were A(H1)pdm09 and 20,880 (44%) were A(H3) viruses. The lineage of 4,679 B viruses was determined and 4,585 (98%) were B/Victoria lineage viruses [1,2].

WHO: World Health Organization.

National Influenza Centres (NICs) in the Region collect influenza virological surveillance data, conduct genetic and antigenic characterisation of viruses and report to The European Surveillance System (TESSy) on a weekly basis. The WHO Collaborating Centres (WHO CC) in London and in Atlanta (at the Centers for Disease Control and Prevention (CDC)) provide NICs with post-infection ferret antisera or other antisera raised against egg and/or cell culture-propagated vaccine/reference viruses for antigenic characterisation or typing/subtyping using haemagglutination inhibition (HAI) assays. WHO CC London also provides a list of reference sequences for the assignment of viruses to haemagglutinin (HA) gene clades/subclades following Sanger or next generation sequencing (NGS) [3]. NICs share representative influenza-positive samples with the WHO CC for in depth antigenic and genetic analyses essential for decision-making at vaccine composition meetings (VCMs).

Fifty Member States of the WHO European Region reported 165,267 influenza virus detections between week 40 2019 through week 20 2020. Relative proportions of circulating influenza A(H3), A(H1)pdm09 and B/Victoria lineage viruses varied between countries [1,4]. Only 24 of the 50 countries reporting influenza detection data contributed virus characterisation data. Of all viruses detected, 2% (2,601/165,267) were antigenically and 2% (3,403/165,267) were genetically characterised ahead of the 2020 southern hemisphere (SH) VCM [1]. Virus characterisation data were used to determine the similarity of circulating viruses to the components of influenza vaccines for the 2019/20 NH influenza season and to assess implications of the COVID-19 pandemic on influenza surveillance and its output.

## Influenza virus characterisation in the WHO European Region in light of the COVID-19 pandemic

The spread of severe acute respiratory syndrome coronavirus 2 (SARS-CoV-2) occurred in March 2020, relatively late in the course of the influenza season in the Region, and the total number of influenza virus detections was comparable to previous seasons. However, the COVID-19 pandemic adversely affected the generation and reporting of virus characterisation data.

**Figure 2:**
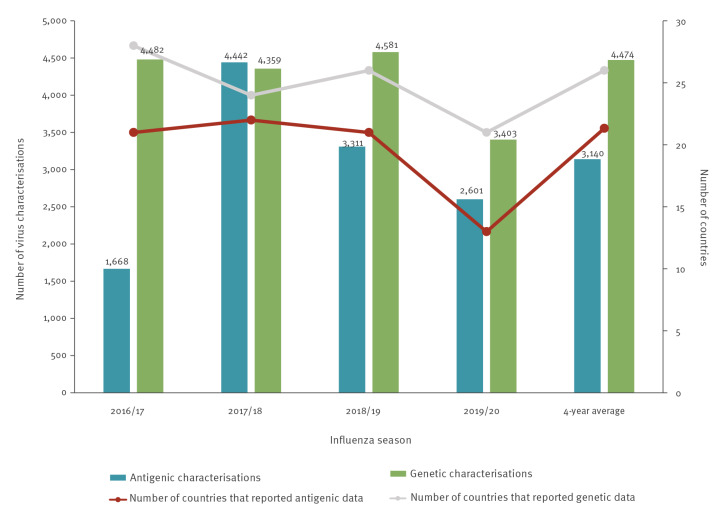
Number of countries reporting influenza virus characterisation data and number of influenza virus characterisations by season, WHO European Region, 2016/17–2019/20

Compared with the previous 4-year averages, a lower number of countries contributed antigenic and genetic data in 2019/20 (13 and 21 vs 21 and 26, respectively) (Figure 2), and the number of antigenic and genetic characterisations decreased by 17% (2,601 vs 3,140) and 24% (3,403 vs 4,474) respectively (Figure 2). The most pronounced decrease was observed in the number of countries reporting antigenic characterisations, possibly reflecting reduced access to laboratory resources and equipment, biosafety concerns or pressure on human resources. Notably, virus characterisation reports effectively stopped in March 2020, and few influenza viruses were detected thereafter, whereas in previous years positive samples were collected and viruses characterised throughout the year.

WHO: World Health Organization.

## Genetic and antigenic analysis of circulating influenza viruses, 2019/20

For specimens collected from week 40 2019 to 20 2020, genetic characterisation data of 3,403 viruses were reported to TESSy by 21 countries and antigenic characterisation data of 2,601 viruses by 13 countries. Table 1 and Table 2 provide the full list of numbers of viruses in each antigenic group and genetic clade, reporting category by week of sample collection.

**Table 1:** Antigenic characteristics of influenza viruses as reported to TESSy by week of sampling, WHO European Region, week 40 2019–week 20 2020 (n = 2,601 viruses characterised in 13 countries)

Year and week	Influenza B				Influenza A							TOTAL
	Yamagata^a^	Victoria^b^			H3					H1		
	B/Phuket/3073/2013-like^c^	No category	B/Colorado/06/2017-like^d^	B/Washington/02/2019-like^e^	A/Switzerland/8060/2017-like	No category	A/Singapore/INF-16-0019/2016-like	A/South Australia/34/2019-like^f^	A/Kansas/14/2017-like^g^	No category	A/Brisbane/02/2018-like^h^	
**2019**												
40	0	0	1	3	0	0	1	0	2	0	2	**9**
41	0	0	3	0	0	1	0	0	3	0	1	**8**
42	0	0	0	0	0	0	0	1	6	0	3	**10**
43	0	0	0	0	0	2	0	1	9	0	3	**15**
44	0	0	0	3	0	0	0	0	13	0	4	**20**
45	0	0	0	5	1	1	1	0	13	0	11	**32**
46	0	0	4	11	2	3	1	1	10	1	8	**41**
47	0	0	2	12	0	2	0	1	64	0	8	**89**
48	0	0	6	13	1	0	1	1	66	3	10	**101**
49	0	1	6	9	0	2	1	1	90	7	15	**132**
50	0	0	12	22	4	6	1	0	75	6	37	**163**
51	0	0	25	27	0	4	2	0	116	11	35	**220**
52	1	0	10	9	1	4	6	0	12	5	22	**70**
**2020**												
1	0	0	2	7	2	2	1	1	2	10	34	**61**
2	0	0	8	13	0	3	0	1	17	12	51	**105**
3	0	0	23	51	0	0	2	0	24	14	72	**186**
4	0	0	21	20	0	0	5	0	36	3	105	**190**
5	1	0	29	25	0	0	11	0	63	17	94	**240**
6	0	0	26	23	0	0	10	0	53	18	75	**205**
7	0	0	16	24	0	0	23	0	39	24	63	**189**
8	0	0	28	8	0	0	12	0	26	14	63	**151**
9	0	0	24	6	0	0	5	0	50	7	69	**161**
10	0	0	15	3	0	0	1	0	33	11	36	**99**
11	0	0	6	5	0	0	2	0	16	5	22	**56**
12	0	0	8	1	0	1	2	0	5	3	10	**30**
13	0	0	5	0	0	0	1	0	4	2	5	**17**
14	0	0	0	0	0	0	0	0	0	0	1	**1**
15	0	0	0	0	0	0	0	0	0	0	0	**0**
16	0	0	0	0	0	0	0	0	0	0	0	**0**
17	0	0	0	0	0	0	0	0	0	0	0	**0**
18	0	0	0	0	0	0	0	0	0	0	0	**0**
19	0	0	0	0	0	0	0	0	0	0	0	**0**
20	0	0	0	0	0	0	0	0	0	0	0	**0**
**Total**	**2**	**1**	**280**	**300**	**11**	**31**	**89**	**8**	**847**	**173**	**859**	**2,601**

TESSy: The European Surveillance System; WHO: World Health Organization.

^a^ Within influenza B Yamagata lineage, no viruses were reported as not belonging to a pre-defined antigenic category.

^b^ For influenza B Victoria lineage, no viruses were reported as being B/Brisbane/60/2008-like.

^c^ Vaccine component in quadrivalent both northern (2019/20 season) and southern (2020 season) hemispheres.

^d^ Vaccine component for use in northern hemisphere 2019–2020 season.

^e^ Vaccine component for the southern hemisphere 2020 season.

^f^ Vaccine component for the southern hemisphere 2020 season.

^g^ Vaccine component for the northern hemisphere 2019–2020 season.

^h^ Vaccine component for both northern (2019–2020 season) and southern (2020 season) hemispheres.

**Table 2:** Genetic characteristics of influenza viruses as reported to TESSy by week of sampling, WHO European Region, week 40 2019–week 20 2020 (n = 3,403 viruses characterised in 21 countries)

Year and week	Influenza B							Influenza A									TOTAL
	Yamagata^a^		Victoria^a^					H1^a^					H3^a^				
	Subgroup not listed	3^b^ _ B/ Phuket/3073/2013	Subgroup not listed	1A(Δ162-164)Β_ B/ Washington /02/2019^c^	No clade	1Α(Δ162-163) _ B/ Colorado/06/2017^d^	1A(Δ162-164) _ B/ Hong Kong/269/2017	Subgroup not listed	6Β.1Α7 _ A/ Slovenia/1489/2019	6Β.1Α1 _ A/ Brisbane/02/2018^e^	6Β.1Α5Α _ A/ Norway/3433 /2018	6Β.1Α5Β _ A/ Switzerland/3330/2018	3C.2a1b +T131K- B _ A/ South Australia/34/19^f^	3C.3a _ A/ Kansas/14/2017^g^	3C.2a1b +T135K _ A/ La Rioja/2202/2018	3C.2a1b +T135K-B _ A/ Hong Kong/2675/19	
2019																	
40	0	0	0	5	0	0	0	0	0	0	3	1	4	7	1	2	**23**
41	0	0	0	1	0	1	0	0	0	0	6	0	10	5	1	1	**25**
42	0	0	0	4	0	1	0	0	0	0	9	0	9	2	1	1	**27**
43	0	1	0	5	0	0	0	0	0	0	6	1	13	4	2	4	**36**
44	0	1	0	9	0	0	0	0	0	0	17	3	12	13	0	7	**62**
45	0	0	0	13	0	0	0	1	0	0	12	8	10	19	2	4	**69**
46	0	0	0	17	0	1	0	1	0	0	27	6	5	17	2	3	**79**
47	0	2	1	32	0	0	0	0	1	1	23	2	24	22	3	9	**120**
48	0	7	1	50	1	1	0	1	0	1	34	1	20	25	2	10	**154**
49	1	2	2	41	0	2	1	0	1	3	40	2	20	46	2	5	**168**
50	0	3	3	63	0	0	0	4	2	0	55	2	15	41	3	4	**195**
51	1	2	0	60	0	7	0	1	2	4	51	1	21	60	11	8	**229**
52	0	1	1	47	0	3	2	1	1	2	56	2	26	53	6	7	**208**
2020																	
1	0	3	2	47	0	1	0	5	1	7	95	3	31	37	3	6	**241**
2	0	3	6	48	0	0	0	4	6	1	123	4	27	39	5	5	**271**
3	0	1	2	92	0	2	0	0	5	2	170	6	25	63	7	7	**382**
4	0	0	4	41	0	0	0	0	3	0	98	3	16	35	11	3	**214**
5	0	1	11	40	0	1	1	0	0	0	92	2	17	46	6	3	**220**
6	0	0	5	41	0	0	1	1	0	3	55	3	20	47	4	4	**184**
7	0	1	2	41	0	1	0	0	1	0	48	2	23	31	5	0	**155**
8	0	0	0	21	0	0	0	0	0	0	26	0	5	18	0	0	**70**
9	0	0	0	19	0	0	0	1	0	2	21	0	4	15	2	1	**65**
10	0	0	0	25	0	0	0	2	0	2	30	0	7	18	1	0	**85**
11	0	0	0	27	0	1	0	0	0	0	14	0	11	12	1	0	**66**
12	0	0	0	15	0	0	0	0	0	0	8	0	7	3	0	0	**33**
13	0	0	0	11	0	0	0	0	0	0	2	0	4	1	0	0	**18**
14	0	0	0	3	0	0	0	0	0	0	0	0	0	0	0	0	**3**
15	0	0	0	1	0	0	0	0	0	0	0	0	0	0	0	0	**1**
16	0	0	0	0	0	0	0	0	0	0	0	0	0	0	0	0	**0**
17	0	0	0	0	0	0	0	0	0	0	0	0	0	0	0	0	**0**
18	0	0	0	0	0	0	0	0	0	0	0	0	0	0	0	0	**0**
19	0	0	0	0	0	0	0	0	0	0	0	0	0	0	0	0	**0**
20	0	0	0	0	0	0	0	0	0	0	0	0	0	0	0	0	**0**
**Total**	**2**	**28**	**40**	**819**	**1**	**22**	**5**	**22**	**23**	**28**	**1,121**	**52**	**386**	**679**	**81**	**94**	**3,403**

TESSy: The European Surveillance System; WHO: World Health Organization.

^a^ For influenza B viruses of the Yamagata lineage, no viruses were reported as not belonging to a predefined genetic clade (‘no clade’), while for influenza B viruses of the Victoria lineage, no viruses of clade 1A without amino-acid deletions, represented by the B/Brisbane/60/2008, were detected. For influenza A(H1) no viruses were reported as ‘no clade’ and no viruses were detected as being part of subclade 6B.1A6, represented by A/Ireland/84630/2018. For influenza A(H3) no viruses were reported with ‘no clade’ and no viruses had a ‘subgroup not listed’.

^b ^Vaccine component in quadrivalent both northern (2019/20 season) and southern (2020 season) hemispheres.

^c^ Vaccine component for the southern hemisphere 2020 season.

^d^ Vaccine component for use in northern hemisphere 2019/20 season.

^e^ Vaccine component for both northern (2019/20 season) and southern (2020 season) hemispheres.

^f^ Vaccine component for the southern hemisphere 2020 season.

^g^ Vaccine component for the northern hemisphere 2019/20 season.

Among A(H1)pdm09 viruses, of the 1,246 that were genetically characterised, 1,121 (90%) belonged to the 6B.1A5A group, moreover, of the 1,032 antigenically characterised, the majority (n = 859; 83%) were similar to the A/Brisbane/02/2018 vaccine virus. However, 173 A(H1)pdm09 viruses were not attributed to any predefined antigenic category, indicative of possible antigenic drift; of these viruses, genetic information was reported for only 48, nine of which had the HA1 N156K amino-acid substitution in antigenic site Sa. Overall, 16% (168/1,049) of A(H1)pdm09 viruses with genetic sequence information were 6B.1A5A-156K.

Of the 1,240 genetically characterised A(H3) viruses, the majority (n = 679; 55%) belonged to clade 3C.3a and were antigenically similar to the NH 2019/20 vaccine virus A/Kansas/14/2017. The remainder belonged to subclade 3C.2a1b and were antigenically distinct [5]. Of the 986 antigenically characterised viruses, most (n = 847, 86%) were characterised as A/Kansas/14/2017-like. The high proportion of viruses antigenically characterised as clade 3C.3a viruses probably reflects issues with characterisation of subclade 3C.2a1b viruses by HAI; 3C.2a1b viruses do not agglutinate red blood cells well and therefore were less tested with HAI [3,6,7].

Of 917 genetically characterised type B viruses, the B/Victoria-lineage accounted for 887 (97%), with 819 (92%) of these belonging to clade 1A(Δ162–164-B) and being antigenically distinct from the clade 1A(Δ162–163) vaccine virus B/Colorado/06/2017. Only 30/917 (3%) of type B viruses were assigned to the B/Yamagata-lineage, and 28 of these were assigned to clade 3, remaining antigenically similar to the B/Phuket/3073/2013 vaccine virus.

## Ethical statement

An ethical approval was not needed for this study, as data are not identifiable back to the patients from whom they originated.

## Discussion

Based on the data, influenza activity in the European Region appears to have ended abruptly in week 13 2020, earlier than previous seasons [1,8,9]. Responses to the COVID-19 pandemic, e.g. changes in access to and utilisation of healthcare and SARS-CoV-2 non-pharmaceutical control measures, such as school closures and social distancing, likely impeded continued surveillance and spread of influenza. This resulted in few influenza viruses being detected after week 13 2020 and, overall, fewer viruses being characterised, despite the obvious efforts from the laboratories under high pressure and overwhelming work load. Redirection of laboratory testing capacities to SARS-CoV-2, with shortages of laboratory supplies and human resources, could also explain the reduced level of influenza virus characterisation.

During the 2019/20 influenza season, the vast majority of influenza A(H3) viruses fell in genetic clade 3C.3a and subclade 3C.2a1b. The 3C.3a viruses were antigenically similar to the recommended 2019/20 NH vaccine virus, while 3C.2a1b viruses were antigenically distinct [5,6]. Most A(H1)pdm09 viruses fell in clade 6B.1A5A (90%) with the majority being antigenically similar to the vaccine virus. However, antigenically distinct viruses with HA1 N156K amino acid substitution were detected. Numbers of viruses in the 6B.1A5A-156K group increased rapidly in many countries simultaneously worldwide, notably in some of those that had A(H1)pdm09 epidemics, resulting in a change of the A(H1)pdm09 vaccine component for the SH 2021 influenza season [5,9]. Of the B/Victoria lineage viruses, accounting for 98% of type B viruses, the vast majority belonged to the Δ162–164-B triple deletion subgroup and were antigenically distinct from the vaccine virus.

Despite circulation of viruses antigenically distinct from vaccine components (i.e. A(H3) subclade 3C.2a1b, B/Victoria-lineage Δ162–164-B and A(H1)pdm09 6B.1A5A-156K viruses) during 2019/20, a moderately good overall level of vaccine effectiveness was observed, notably for type B and A(H1)pdm09 viruses [10]. The issue of poor recognition of circulating A(H3) viruses by immune responses to egg-propagated vaccine virus remained [11].

Data from the 2020 SH influenza season show that circulation of influenza viruses was extremely limited in the SH winter and also elsewhere for the NH inter-seasonal period [5,9,12]. Similar low levels of influenza might be expected in the WHO European Region in the 2020/21 season, if COVID-19-related public health measures are implemented. However, co-circulation of both influenza and SARS-CoV-2 viruses is possible, and should warrant resource-related and operational prioritisation efforts to ensure that continued evidence-based decisions can be made at WHO influenza VCMs. In either scenario, NICs will be challenged to ensure collection of representative specimens for influenza virus detection and subsequent virus characterisations with laboratory capacities being divided between influenza and SARS-CoV-2 surveillance [13]. The European Centre for Disease Prevention and Control (ECDC) and WHO Regional Office for Europe have issued joint interim guidance on what approaches should be used to maintain influenza surveillance during the winter period with the ongoing COVID-19 pandemic [13].

In terms of way forward, NICs play crucial roles in surveillance of seasonal influenza and zoonotic events and are responsible for arranging the essential shipments of representative specimens to the WHO CC to ensure there are sufficient data for making VCM recommendations. With increasing number of avian influenza outbreaks and continued evolution of influenza viruses in swine, there is also need for maintained vigilance in public health laboratories to ensure detection of zoonotic events for pandemic preparedness purposes [14,15]. In the 2020/21 season, efforts are needed to ensure maintenance of influenza surveillance, but also to support COVID-19 surveillance to understand SARS-CoV-2 transmission and inform national responses to the pandemic.

## WHO European Region Influenza Surveillance Network affiliations of authors

**Austria**: Monika Redlberger-Fritz, monika.redlberger@meduniwien.ac.at, National Influenza Centre for Austria, Center for Virology, Medical University Vienna, Judith Aberle, judith.aberle@meduniwien.ac.at, National Influenza Centre for Austria, Center for Virology, Medical University Vienna

**Denmark**: Ramona Trebbien, RATR@ssi.dk, National Influenza Center, Statens Serum Institut, Denmark, Jakob Nybo Nissen, JANN@ssi.dk, National Influenza Center, Statens Serum Institut, Denmark.

**Finland**: Niina Ikonen, Finnish Institute for Health and Welfare, Department of Health Security, niina.ikonen@thl.fi, Anu Haveri, Finnish Institute for Health and Welfare, Department of Health Security, anu.haveri@thl.fi

**Germany**: Ralf Dürrwald DuerrwaldR@rki.de

**Greece**: Georgia Gioula ggioula@auth.gr, National Influenza Centre for Northern Greece, Maria Exindari, mexidari@auth.gr National Influenza Centre for Northern Greece, Athanasios Kossyvakis, akossivakis@pasteur.gr, Hellenic Pasteur Institute, National Influenza Reference Laboratory of Southern Greece, Athens, Andreas Mentis, mentis@pasteur.gr, Hellenic Pasteur Institute, National Influenza Reference Laboratory of Southern Greece, Athens

**Ireland**: Linda Dunford, linda.dunford@ucd.ie, National Virus Reference Laboratory, University College Dublin, Ireland, Lisa Domegan, lisa.domegan@hse.ie, Health Service Executive, Health Protection Surveillance Centre, Dublin, Ireland.

**Italy**: Maria Rita Castrucci, mariarita.castrucci@iss.it, Istituto Superiore di Sanità, Department of Infectious Diseases, Rome, Italy, Simona Puzelli, simona.puzelli@iss.it, Istituto Superiore di Sanità, Department of Infectious Diseases, Rome, Italy

**Latvia**: Natalija Zamjatina, National Influenza Centre for Latvia, natalija.zamjatina@aslimnica.lv, Gatis Pakarna, National Influenza Centre for Latvia, gatis.pakarna@aslimnica.lv

**Lithuania**: Algirdas Griskevičius, algirdas.griskevicius@nvspl.lt, National Public Health Surveillance Laboratory, Asta Skrickiene, Centre for Communicable Diseases and AIDS, asta.skrickiene@ulac.lt

**Luxembourg**: Guillaume Fournier Guillaume.Fournier@lns.etat.lu, National Health Laboratory, Luxembourg, Joel Mossong Joel.Mossong@lns.etat.lu, National Health Laboratory, Luxembourg

**Netherlands**: Adam Meijer, adam.meijer@rivm.nl, National Institute for Public Health and the Environment, Bilthoven location of the Dutch National Influenza Centre, Bilthoven, The Netherlands, Ron Fouchier, r.fouchier@erasmusmc.nl, Erasmus University Medical Center, Rotterdam location of the Dutch National Influenza Centre, Rotterdam, The Netherlands

**Norway**: Karoline Bragstad, Karoline.Bragstad@fhi.no, Olav Hungnes, olav.hungnes@fhi.no, Norwegian Institute of Public Health

**Portugal**: Raquel Guiomar, Raquel.guiomar@insa.min-saude.pt, National Influenza Reference Laboratory, Infectious Diseases Department, National Institute of Health Dr Ricardo Jorge, Lisbon, Portugal, Pedro Pechirra, Pedro.Pechirra@insa.min-saude.pt, National Influenza Reference Laboratory, Infectious Diseases Department, National Institute of Health Dr Ricardo Jorge, Lisbon, Portugal

**Romania**: Mihaela Lazar, lazar.mihaela@cantacuzino.ro, National Influenza Center, “Cantacuzino” National Military-Medical Institute for Research and Development, Bucharest, Romania, Cherciu Carmen Maria, cherciu.maria@cantacuzino.ro National Influenza Center, “Cantacuzino” National Military-Medical Institute for Research and Development, Bucharest, Romania

**The Russian Federation**: Andrey Komissarov, a.b.komissarov@gmail.com, NIC, FSBI “Research Institute of Influenza named after A.A. Smorodintsev” Ministry of Health of the Russian Federation, St.-Petersburg, Daria Danilenko, Smorodintsev Research Institute of Influenza, daria.baibus@gmail.com. Elena Burtseva, NIC, FSBI “N.F. Gamaleya NRCEM” Ministry of Public Health of the Russian Federation, Moscow, the Russian Federation, elena-burtseva@yandex.ru

**Slovakia**: Elena Tichá, elena.ticha@uvzsr.sk, National Influenza Centre for Slovakia Edita Staronova, edita.staronova@uvzsr.sk, National Influenza Centre for Slovakia

**Slovenia**: Katarina Prosenc, katarina.prosenc@nlzoh.si, National Laboratory for Health, environment and food, Nataša Berginc, natasa.berginc@nlzoh.si, National Laboratory for Health, environment and food

**Spain**: Francisco Pozo, pacopozo@isciii.es, NIC-Madrid, Instituto de Salud Carlos III, Majadahonda Inmaculada Casas, icasas@isciii.es, NIC-Madrid, Instituto de Salud Carlos III, Majadahonda

**Sweden**: Mia Brytting, mia.brytting@folkhalsomyndigheten.se, Public Health Agency of Sweden

**Switzerland**: Ana Rita Gonçalves, AnaRita.GoncalvesCabecinhas@hcuge.ch, National Reference Centre for Influenza, Laboratory of Virology, Geneva University Hospitals, Geneva

**Ukraine**: Iryna Demchyshyna, irad@i.ua, National Influenza Centre for Ukraine

**United Kingdom**: Angie Lackenby, Angie.Lackenby@phe.gov.uk, National Infection Service, Public Health England, United Kingdom, Catherine Thompson, Catherine.Thompson@phe.gov.uk, National Infection Service, Public Health England, United Kingdom, Rory N Gunson, Rory.Gunson@ggc.scot.nhs.uk, West of Scotland Specialist Virology Centre, Scotland, Samantha J Shepherd, Samantha.Shepherd@ggc.scot.nhs.uk, West of Scotland Specialist Virology Centre, Scotland, Catherine Moore, catherine.moore2@wales.nhs.uk, Public Health Wales Specialist Virology Centre, Wales, Simon Cottrell, Simon.Cottrell@wales.nhs.uk Public Health Wales Communicable Disease Surveillance Centre and Vaccine Programme

**European Centre for Disease Prevention and Control**: Angeliki Melidou Angeliki.melidou@ecdc.europa.eu, Cornelia Adlhoch cornelia.adlhoch@ecdc.europa.eu, Oksana Martinuka oksanamartinyk@gmail.com, Pasi Penttinen Pasi.penttinen@ecdc.europa.eu, Lucia Pastore Celentano lucia.pastore.celentano@ecdc.europa.eu

**WHO Regional Office for Europe**: Dmitriy Pereyaslov pereyaslovd@who.int, Miriam Sneiderman sneidermanm@who.int, James Fielding James.Fielding@vidrl.org.au, Richard Pebody pebodyr@who.int

**WHO Collaborating Centre for reference and research on influenza, Crick Worldwide Influenza Centre, The Francis Crick Institute, London, United Kingdom**: John McCauley John.McCauley@crick.ac.uk, Rodney Daniels Rod.Daniels@crick.ac.uk
